# Whether the Weather Drives Patterns of Endemic Amphibian Chytridiomycosis: A Pathogen Proliferation Approach

**DOI:** 10.1371/journal.pone.0061061

**Published:** 2013-04-17

**Authors:** Kris A. Murray, Lee F. Skerratt, Stephen Garland, Darren Kriticos, Hamish McCallum

**Affiliations:** 1 EcoHealth Alliance, New York, New York, United States of America; 2 Tropical Medicine and Rehabilitation Sciences and the Amphibian Disease Ecology Group, School of Public Health, James Cook University, Townsville, Queensland, Australia; 3 CSIRO Ecosystem Sciences and the Cooperative Research Centre for National Plant Biosecurity, Canberra, Australia; 4 School of Environment, Nathan Campus, Griffith University, Queensland, Australia; Tuscia University, Italy

## Abstract

The pandemic amphibian disease chytridiomycosis often exhibits strong seasonality in both prevalence and disease-associated mortality once it becomes endemic. One hypothesis that could explain this temporal pattern is that simple weather-driven pathogen proliferation (population growth) is a major driver of chytridiomycosis disease dynamics. Despite various elaborations of this hypothesis in the literature for explaining amphibian declines (e.g., the chytrid thermal-optimum hypothesis) it has not been formally tested on infection patterns in the wild. In this study we developed a simple process-based model to simulate the growth of the pathogen *Batrachochytrium dendrobatidis* (*Bd*) under varying weather conditions to provide an *a priori* test of a weather-linked pathogen proliferation hypothesis for endemic chytridiomycosis. We found strong support for several predictions of the proliferation hypothesis when applied to our model species, *Litoria pearsoniana,* sampled across multiple sites and years: the weather-driven simulations of pathogen growth potential (represented as a growth index in the 30 days prior to sampling; GI_30_) were positively related to both the prevalence and intensity of *Bd* infections, which were themselves strongly and positively correlated. In addition, a machine-learning classifier achieved ∼72% success in classifying positive qPCR results when utilising just three informative predictors 1) GI_30_, 2) frog body size and 3) rain on the day of sampling. Hence, while intrinsic traits of the individuals sampled (species, size, sex) and nuisance sampling variables (rainfall when sampling) influenced infection patterns obtained when sampling via qPCR, our results also strongly suggest that weather-linked pathogen proliferation plays a key role in the infection dynamics of endemic chytridiomycosis in our study system. Predictive applications of the model include surveillance design, outbreak preparedness and response, climate change scenario modelling and the interpretation of historical patterns of amphibian decline.

## Introduction

Amphibian chytrid fungus, *Batrachochytrium dendrobatidis* (hereafter *Bd*), is the infective agent of the pandemic amphibian disease chytridiomycosis [1], which has been implicated in global amphibian declines and extinctions [2]–[4]. Seasonal and elevational variation in the presence and prevalence of *Bd* has long implicated temperature as an important mechanistic feature of chytridiomycosis epidemiology, with infections and mortalities more frequently observed in cooler climes and periods [1], [5]–[9]. Laboratory cultures confirm temperature dependent growth; *Bd* can survive freezing in media used for cryo-archiving (but stops growing), grows slowly at low temperatures, grows well between 17–25°C with a peak at 23°C, but growth stops at 28°C and death is observed at 30°C [5], [10]–[12].

The above growth studies and *in vitro* thresholds have been used by many researchers to retrospectively interpret patterns of infection (and in some cases declines) [13] with respect to the optimal temperature range over which *Bd* grows in the laboratory (e.g., [6], [9], [14]). However, despite the tremendous attention this topic has received in the *Bd* literature, quantitative process-based models underlying the proposed relationships between pathogen growth, disease prevalence, intensity of infection, mortality and ultimately population decline and extinction remain poorly explored (but see [15], for simulation modelling of transmission dynamics and an address of historical amphibian declines, respectively). For example, knowledge on *Bd’s* responses to temperature has not been explicitly used for the prediction of disease outbreaks in wild settings, which could potentially be valuable for the design of surveillance and management actions.

This may in part be due to the complexity observed in many systems, as a large number of variables other than temperature have also been reported to influence patterns of *Bd* infection. For example, presence and prevalence of *Bd* in wild hosts reportedly varies with species, life-stage, year and body size [5]–[9], [17], [18]. Detecting a signature of climate (e.g., seasons) and weather (i.e., shorter-term fluctuations in climatic variables such as daily maximum temperature) in the epidemiology of chytridiomycosis is thus complicated not only by the study system and metric used (i.e., infection status, infection intensity, host mortality, population decline/extirpation, extinction) but also by species-specific responses (e.g., differential susceptibility/immunity), invasion history and disease dynamics [3], [15], [16], [19], [20] and the numerous other factors that may influence infection (e.g., host density, breeding behaviour, predator fluctuations, and so on). The ‘thermal-optimum hypothesis’ [13], for example, as applied to the study of population declines (see also [16]) does not explicitly include the potential effects of other climatic drivers (some of which may nevertheless co-vary with temperature), such as moisture availability, that have also been correlated with *Bd* occurrence or prevalence in field-based disease investigations (e.g., [6], [9], [20]–[22]).

Herein we devise a novel test of what we term the ‘weather-linked *Bd* proliferation hypothesis’ for endemic chytridiomycosis. Under this hypothesis, the dynamics of *Bd* infections are strongly influenced by weather-driven pathogen growth within the population. In contrast to previous studies that have descriptively employed ‘thermal optima’ type models to help interpret patterns of infection, our *a priori* aim was to empirically test our hypothesis by quantifying the contribution that simulated weather-linked *Bd* proliferation makes to the dynamics of chytridiomycosis in wild, endemically infected amphibians. To do this, we developed a corresponding ‘weather-linked *Bd* proliferation model’ to simulate the growth of *Bd* under varying conditions. Our model is thus process-based and predictive, based primarily on eco-physiological data and narrowly focussed on an initial step in the epidemiological chain, pathogen growth. In this context, it is important to recognise that simple microparasite compartmental models that classify individuals as susceptible, infected or resistant (Anderson and May 1979) are of limited value for chytridiomycosis. The parasite burden on the individual host appears to be of critical importance in relation to both pathogenicity and transmissibility and therefore has characteristics used to model macroparasites [15].

Three testable predictions follow from the ‘weather-linked *Bd* proliferation hypothesis’ that together form the foundation of this study. Prediction 1: simulated *Bd* growth should be positively related to individuals’ intensities of infection across space and time because frogs represent an ectothermic growth medium for *Bd* [23]–[25]; Prediction 2: mean intensity of infection in individuals should be positively related to the disease prevalence within a population because transmission/infection dynamics will be dependent upon the number of dispersing zoospores present in the population; and Prediction 3: simulated *Bd* growth should thus also be useful for predicting, and be positively related to, the probability of an individual being infected at the time of sampling. In practice, our predictions were tested in reverse order throughout for increased conservatism and broader applicability given previous studies and limitations of the available data (see “Prediction testing overview” in Methods for further detail). In resolving the utility of the weather-linked *Bd* proliferation hypothesis for the prediction of *Bd* infection patterns, we provide a novel means for improving current surveillance (including tailoring sampling for *Bd* to sites and times of enhanced detectability) and management actions (such as outbreak preparedness and response; for example, under future climate change scenarios).

## Methods

### Weather-linked Bd Proliferation Model

The process-based proliferation model was developed in the program CLIMEX [26], [27]. The parameters used in the model together define the species’ response to multiple climatic variables, including temperature and soil moisture availability. The soil moisture index integrates the effects of rainfall and evapotranspiration, simulating effective rainfall. In concert with temperature, this has been shown to be closely associated with habitat-defining factors such as vegetation [27]. A weekly growth index (GI_W_) determines the potential for the *Bd* population to increase during favourable periods for a given climate dataset. The GI_W_ (range 0–1) was calculated on interpolated weather data for each site and sample (source: Australian Bureau of Meteorology Data-drill database; http://www.longpaddock.qld.gov.au/silo/). It is the product of the temperature and soil moisture indices (TI*MI), which are each formulated in accord with Shelford’s law of tolerance [28], which posits that the success of an organism is based on a complex set of conditions such that each individual or population has a minimum, maximum, and optimum for each factor or combination of factors that determine success. The TI_W_ and MI_W_ together describe the range over which growth is possible and the range over which growth is optimal. The combination of the temperature and soil moisture indices accords with the principal that the resource in shortest supply provides the greatest limitation on the growth of a species [29]. CLIMEX (and its GI_W_) has been used extensively in pest risk analysis and biological control studies and has been useful in modelling the growth, distribution and impacts of numerous insects, plants and vertebrates [27], [30].

Temperature parameters were derived from experiments presented in the literature. We first plotted a ‘thermal performance curve’ of the maximum growth rate (increase in optical density value per day of growth) observed in the data of Piotrowski et al. [12]. We fitted these data with a quartic polynomial function (Figure A1a in Appendix 1 in Materials S1), which showed a good fit with high predictive value (R^2^ = 0.93), was biologically realistic (performance curve shaped) and was minimally complex (minimum parameters) [31] (for similar approaches, see [16], [32]). CLIMEX uses a plateau-shaped thermal function to approximate the typical quadratic response function such as that observed here. The curve is defined by the terms T_0_ (growth stops), T_1_ and T_2_ (between which growth is optimal) and T_3_ (growth stops). We estimated T_0_ and T_3_ from where the fitted polynomial function intersected the x-axis to the nearest half degree (3°C and 29°C, respectively). The optimal performance temperature range was estimated by combining observations from Piotrowski et al. [12] and Woodhams et al. [33]. Lower optimal temperature (T_1_) was adjusted to 10°C to accord with Woodhams et al. [33] who demonstrate that *Bd* maintains relatively high population growth at low temperatures via life-history trade-offs, which see increased zoospore production as sporangium maturation rate decreases. The upper optimal temperature (T_2_) was set to 25°C to accord with Piotrowski et al. [12] (see Figure A1a in Appendix 1 in Materials S1 for further details). Our model assumes that temperature-dependence observed in media translates to *Bd* growth in the skin of amphibian hosts. This is a reasonable assumption given that amphibians are ectothermic; however, it is possible that some factors that could not be included in our model, such as temperature-dependent host responses or immunity (e.g., [32]), may also influence the growth of *Bd* in skin. We further assume that growth rate at a constant temperature is an adequate predictor of the growth rate in the environment at an equivalent mean temperature.

Little information is available regarding *Bd’s* response to rainfall/soil moisture. Occurrence patterns indicate that sufficient rainfall is an important factor governing *Bd* distribution [20], [22] and Kriger et al. [21] report higher prevalence and intensity of infections at sites with higher rainfall. We regarded this as an *a priori* reason to suspect that moisture availability plays a key role in *Bd* proliferation. Potential mechanisms for this may include direct effects of moisture on the proliferation of *Bd* on skin, perhaps by enhancing intra-host transmission of infective zoospores which result in self reinfection, and/or by enhanced inter-host transmission by route of the aquatic zoospore [34]–[38]. In the absence of further information that can be used directly to parameterise the model, a feature of CLIMEX is that it enables the user to infer a species’ average response to moisture availability based on knowledge of its geographic pattern or abundance [27]. Soil moisture parameters were thus estimated by adjusting the ‘temperate species' template in CLIMEX via iterative fitting, which in pilot analyses produced results that were highly consistent with the distribution of *Bd* both in Australia [20], [39], [40] (Figure A1b in Appendix 1 in Materials S1) and globally (Figure A1c in Appendix 1 in Materials S1) [41] in combination with the thermal parameters outlined above. Such iterative fitting is standard procedure when building CLIMEX models under such circumstances [27], but due to this correlative component of the model as applied here it is important to distinguish our approach from purely mechanistic approaches that are sometimes also referred to as process-based (see e.g., [42]). The CLIMEX model as applied to modelling the potential distribution of *Bd* globally is the subject of ongoing research (K.A.M et al. unpubl. data) and is not presented herein (but see Figure A1c in Appendix 1 in Materials S1).

### Competing Predictor Variables

In addition to the proliferation model predictions, we investigated eight potentially informative climatic variables at four temporal scales: T.max_X_ (maximum temperature), T.min_X_, Rain_X_, Evap_X_ (Class A pan evaporation), Radn_X_ (short-wave radiation, proxy for UV), VP_X_ (vapour pressure), RH.maxT_X_ (relative humidity at maximum temperature), RH.minT_X_ and FAO56_X_ (potential evapotranspiration), where x is the number of days prior to sampling over which an average is calculated (here, x = 1, 7, 15, 30 days). The various temporal intervals were chosen to reflect the uncertainty as to the time span over which each variable may impact infection. The daily weather information was derived from the Australian Bureau of Meteorology Data Drill facility; http://www.longpaddock.qld.gov.au/silo/). Since the climate data in this study come from interpolated sources, there is an obvious limitation to our analyses in that frogs may not be experiencing the same weather conditions as those estimated for grid station points. Reasons for a mismatch are many but might be primarily due to behavioural characteristics of the species [18], the modifying effect of microclimates [42] and topographic complexity [43]. Nevertheless, we expect that the interpolated weather data are suitably indicative of the conditions and their fluctuations experienced by *Bd* on frogs in the field. Other variables considered in the analyses were year, month, season, site, sex and body size (SUL; snout-urostyle length).

### Infection Pattern Surveys

**Figure 1:**
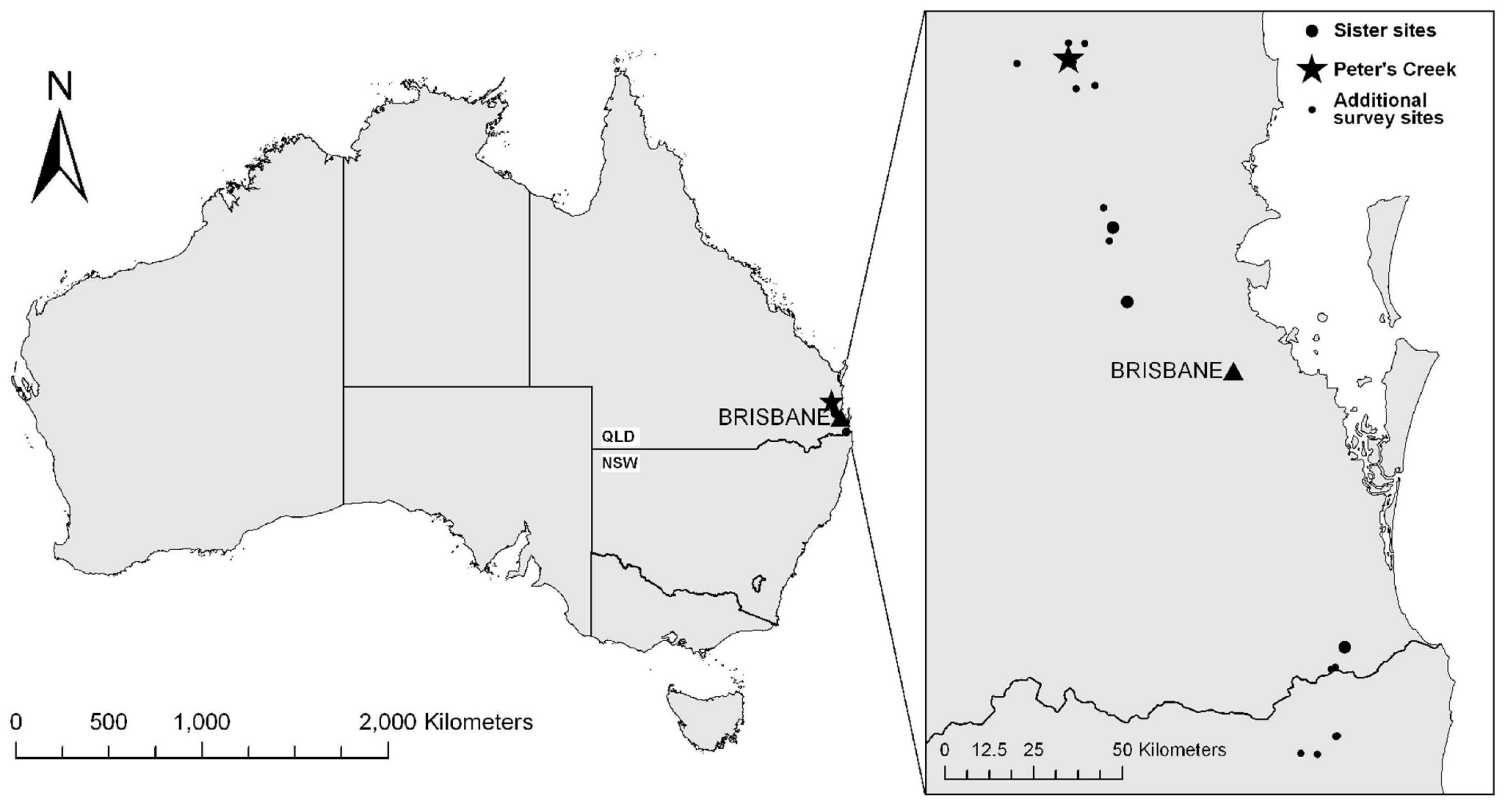
Map of the study region, showing the four main study sites in South-east Queensland as well as the additional survey sites in Queensland and New South Wales, Australia.

We captured all frogs encountered at four main study sites sampled during Spring and Summer (when most frogs are active in subtropical Australia) over three consecutive years (August 2006– March 2009). The main field site (Peter’s Creek) is that used and described in Murray et al. [19]. In addition to the Peter’s Creek study site, three ‘sister’ sites were sampled monthly in the first and second study seasons in other rainforested regions of South-east Queensland. An extent of infection survey was also undertaken at a further 20 sites (Figure 1). All field sites sampled during the study were situated well within the known distribution of *Bd* in Australia and are considered environmentally highly suitable for the persistence of chytridiomycosis [20], [39]. The region is subtropical with warm, wet summers (mean max. temp. ∼27°C; mean rainfall ∼780 mm) and cooler, dryer winters (mean max. temp. ∼19.5°C; mean rainfall ∼250 mm) (Australian Bureau of Meteorology records for Maleny; 425 m asl).

#### Study species

While surveys and analyses were conducted for multiple frog species (see Table A2 in Appendix 2 in Materials S1), sampling was most comprehensive for a single ‘model’ species, *Litoria pearsoniana,* which forms the focus of this study. *L. pearsoniana* is a small, stream-breeding treefrog restricted to forested areas of southeast Queensland and northern New South Wales, Australia. It is listed as vulnerable under the Queensland Nature Conservation (Wildlife) Regulation 2006 and near threatened and ‘in significant decline…. in part due to chytridiomycosis’ by the IUCN [44]. Chytridiomycosis-associated mortality and reductions in apparent survival have been described in this species [5], [19]. Chytridiomycosis does not appear to influence detectability of this species in encounter surveys, which means that sampling of the population is likely to approximate random sampling with respect to disease status [19], [45].

#### Sampling for *Bd*

When encountered, frogs were captured in plastic freezer bags, weighed and removed by hand for examination, ensuring a fresh pair of plastic gloves was worn to process each frog. For sampling infection with *Bd*, frogs were swabbed (10 strokes on each of the feet, thighs and hands, and on the left, centre and right ventral surfaces of the body) with a sterile swab (MW 100–100, Medical Wire and Equipment, Bath). Swabs were stored in the field on ice and later at room temperature [24]. After swabbing, frogs were measured (snout-urostyle length [SUL]), inspected for clinical signs of infection (e.g., erythema of ventral surfaces and digits) and released at the point of capture.

### Diagnostic Methods

We analysed swabs in the laboratory for the presence of *Bd* with the TaqMan real-time qPCR protocol [46] and included an internal positive control to signal amplification inhibition [24]. Hyatt et al. [24] detail the properties of the test including sensitivity, specificity, limitations, and comparisons with other methods.

### Prediction Testing Overview

qPCR provides a quantitative estimate of the amount of *Bd* material (zoospore equivalent count) on the swab and evidence suggests that this may broadly provide an index of an individual’s intensity of infection [23]–[25]. However, because infection intensity has not yet been rigorously tested as being suitable for this purpose and is known to vary in infected frogs with poorly studied factors such as stage in the sloughing cycle [47], we chose to focus the bulk of our exploratory analyses (presented in the Materials S1 Appendices) to the infection status of individuals (i.e., positive or negative for *Bd* in a triplicate qPCR test).

We thus worked backwards through our three predictions, starting with a test of Prediction 3, which states that simulated *Bd* growth should be useful for predicting, and be positively related to, the probability of an individual being infected at the time of sampling. We then more conservatively employed the estimates of infection intensity (zoospore equivalent) to test Prediction 2 that infection intensity of infected individuals should be broadly related to infection prevalence within the sampled population (these are statistically independent as confounding zeros of negative infections at the site level are excluded). Finally, we tested Prediction 1 directly by applying our best model from the test of Prediction 3 to characterise the relationship between simulated pathogen proliferation and infection intensity. This prediction-based approach ensured adequate testing of the underlying mechanisms of our hypothesis while at the same time giving due focus to the problem of predicting infection status, which we felt was a more straightforward prediction problem than infection intensity (a robust binary classification as opposed to a regression model on what has so far been a poorly explored source of continuous data). In addition, our results remain relevant to a broader literature in which disease prevalence is typically reported using a range of diagnostic methods, whereas infection intensity is only explored in studies using qPCR.

### Overview of Statistical Methods

For the full dataset, we were interested in examining the effects of up to 38 variables (6 individual trait variables+the eight climatic variables at four temporal scales), which we knew *a priori* to be inter-correlated to varying degrees. Given the potential complexity of models confronting these data, we broke the analyses down into several stages, all of which are documented in Appendices 2–5 in Materials S1. First, we performed preliminary analyses on data from the main Peter’s Creek study site (a multi-species dataset) in which we used binary logistic regression to model infection as a function of species, year and season. Next, we employed a non-parametric, tree-based approach to determine a reasonable structure and identify the most important variables for subsequent analyses on the larger dataset containing all of the variables of interest [48], [49]. This amounted to a ‘filtering’ process that allowed increasing data simplification without compromising the detection of important factors influencing infection along the way. The final ‘clean’ product was a single-species/single-sex/multi-site dataset (male *L. pearsoniana*) retaining only the most pertinent climatic predictors (see Appendices 2–5 in Materials S1 for further detail). We then supplemented the predictors encoding prevailing weather information with the single metric from our weather-linked *Bd* proliferation model (GI_W_) and assessed predictive performance. We used GI_W_ averaged over the 30 days prior to sampling for analyses (GI_30_).

Tree-based methods are useful when variable interactions and complex, non-linear relationships between predictor variables and the response are to be expected [49], [50]. We used the package ‘randomForests’ implemented in R [51], [52] to run Random Forests (RF) models on the data. RF is an extension of traditional single-tree methods from the field of machine learning that combines the predictions of hundreds or thousands of trees to improve prediction stability and accuracy and better cope with bias and inter-correlation among variables. An in-depth discussion of the RF algorithm, associated metrics and uses of RF in ecology is provided by Cutler et al. [53]. Variable importance measures were used for our ‘pruning’ (variable selection) process and partial dependence plots were used to visualise the relationships between the predictors and the response (infection) [54]. Unlike some other statistical frameworks, RF does not produce *P* values, confidence intervals or regression coefficients. A demonstration of the RF method with reference to the logistic regression described above is presented in Appendix 3 in Materials S1 (see Results). Generalised linear models were also developed to confirm the significance of variables remaining in the pruned RF models (Appendix 6 in Materials S1).

### Ethics Statement

This study was approved under University of Queensland Animal Ethics permit SIB/144/06/ARC, Queensland Parks and Wildlife permits (WITK037994406, WISP03806206, TWB/21/2006, TWB/34/2006) and New South Wales Department of the Environment, Climate Change and Water permit S12742.

## Results

### Preliminary Analyses

#### The effect of species, season, year and sex

During the preliminary analyses, several variables were found to significantly influence the probability of an individual returning a positive result. Briefly, there was a significant three-way interaction between species, season and year. We thus analysed the four most well sampled species in detail separately (Figure A2 in Appendix 2 in Materials S1). A trial run testing the efficacy of the RF modelling framework was largely complementary to the results presented in Appendix 2; there was a clear effect of species, year and season on infection (Figure A3 in Appendix 3 in Materials S1). Since the majority of data came from a single species, *L. pearsoniana,* further analyses were restricted to this “model” species. In the extent of infection survey encompassing 20 additional sites, 100% of *L. pearsoniana* populations were infected with *Bd* (Appendix 4 in Materials S1). A significant effect of sex (also seen in Appendix 3 in Materials S1) was next found to influence infection in *L. pearsoniana* (Appendix 5 in Materials S1). Since most of the data came from male frogs, females and juveniles were excluded for the core analyses presented below. Subsequent results thus apply only to this sub-sample.

### The Effect of Rainfall, Humidity and Temperature

The final dataset on male *L. pearsoniana* from four main study sites comprised 1072 captures, representing roughly half of all captures in this study (see Appendices 2–5 in Materials S1). In an initial ‘saturated’ RF model developed to predict infection status of individual frogs in the dataset and informed by four categorical variables (month, year, season, site) and all raw climatic predictors (i.e., not including the predictions from the proliferation model, GI_30_) the estimate of classification error rate (the out-of-bag or OOB estimate; an internally derived measure of model predictive performance) was intermediate at 32.9%, meaning that the overall percentage of cases correctly classified (PCC) was 67.1%. Specificity (% of negative tests correctly classified) was 62.9%, while sensitivity (% of positive tests correctly classified) was higher at 71.5% (Kappa = 0.343, 95%CI = 0.287–0.399, where Kappa = 0 represents prediction no better than random while 1 represents perfect prediction). This model represents the baseline predictive performance from which further refined models, including those incorporating the predictions of the process-based proliferation model (GI_30_), can be judged.

**Figure 2:**
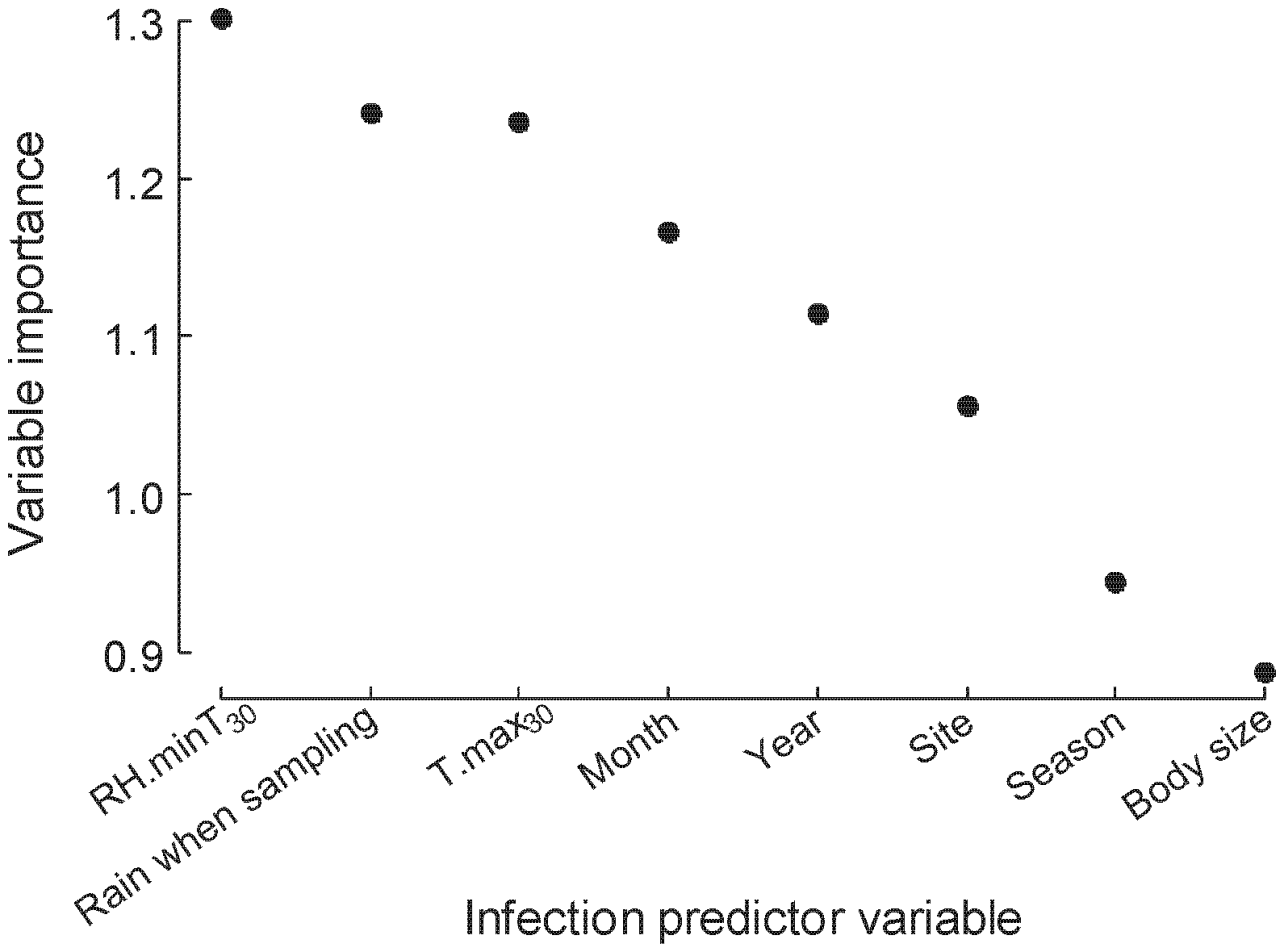
Variable importance plot from the Random Forest (RF) framework for predicting infection status in adult male *Litoria pearsoniana* captured at four study sites across three years of study (2006–2009). To assess importance of each variable: after growing the kth tree, the values of the target variable among all out-of-bag (OOB) cases are randomly permuted and the OOB cases are run down the tree. The decrease in the number of votes for the correct class due to permuting is averaged over the forest. RH.minT_30_ is the average maximum relative humidity in the 30 days prior to sampling, Rain when sampling is the amount of rain (in mm) on the day swabs were taken in the field, T.max_30_ is the average maximum temperature in the 30 days prior to sampling, Year is the year in which samples were taken, Body size is measured as snout-urostyle length (SUL; measured in mm).

Due to the large number of variables with minimal predictive value, we refined this initial model retaining only the best four continuous predictors as indicated by the variable importance metric in RF (RH.minT_30_, SUL, rain on the day of sampling, T.max_30_) together with the original categorical variables (month, year, season, site). This refined model achieved equivalent predictive results (PCC = 68.6%; Specificity = 66.0%, Sensitivity = 71.3%; Kappa = 0.372, 95%CI = 0.317–0.428), indicating no loss of important information via pruning. A plot of variable importance is shown in Figure 2. It can be seen that, for this subgroup, the continuous climatic variables outperformed the categorical and individual trait (SUL) variables in terms of predictive power, suggesting that variation in infection between years, seasons, months and sites could be driven by changes in the raw climatic variables.

**Figure 3:**
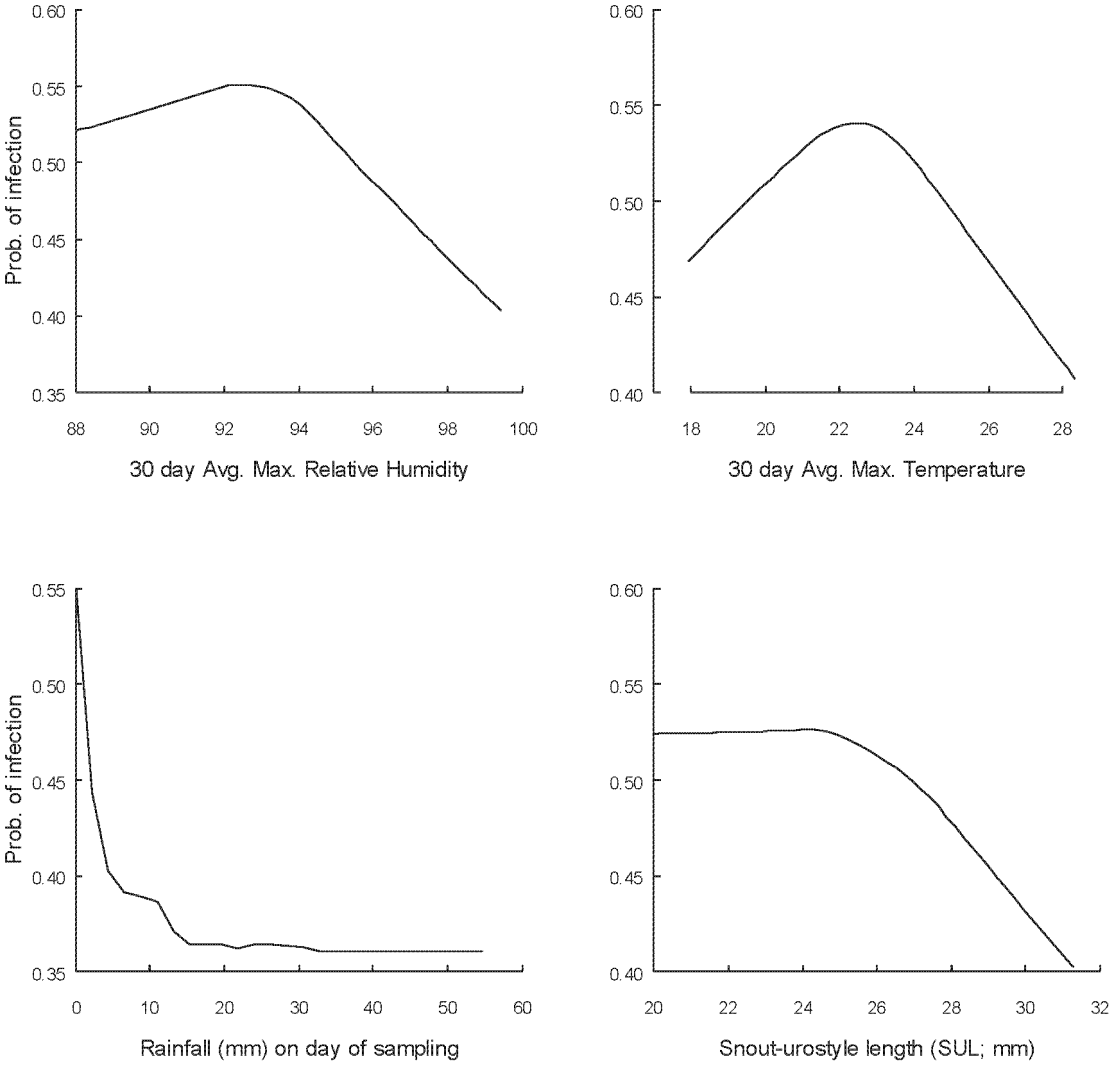
Partial dependence plots from the Random Forest (RF) framework showing the relationship between probability of *Bd* infection in adult male *Litoria pearsoniana* and each of the continuous variables included in the pruned RF model. Sampling was conducted across four sites and three years of study (2006–2009). Response lines are lowess smoothers.

Partial dependence plots describing the relationships between the three most important predictors (all climatic and continuous) and probability of infection are shown in Figure 3. Infection probability was highest when average maximum relative humidity in the preceding 30 days was <95%, when average maximum temperature in the preceding 30 days was in the range 20–25°C and when there was no rainfall on the day of sampling. Very high average maximum humidity, high average maximum temperatures and rain on the day of sampling all decreased the probability of sampled frogs returning a positive PCR result. Probability of infection was also influenced by body size; at large body sizes (SUL >27 mm) there was a decrease in the probability of a frog being infected (Fig. 3).

**Figure 4:**
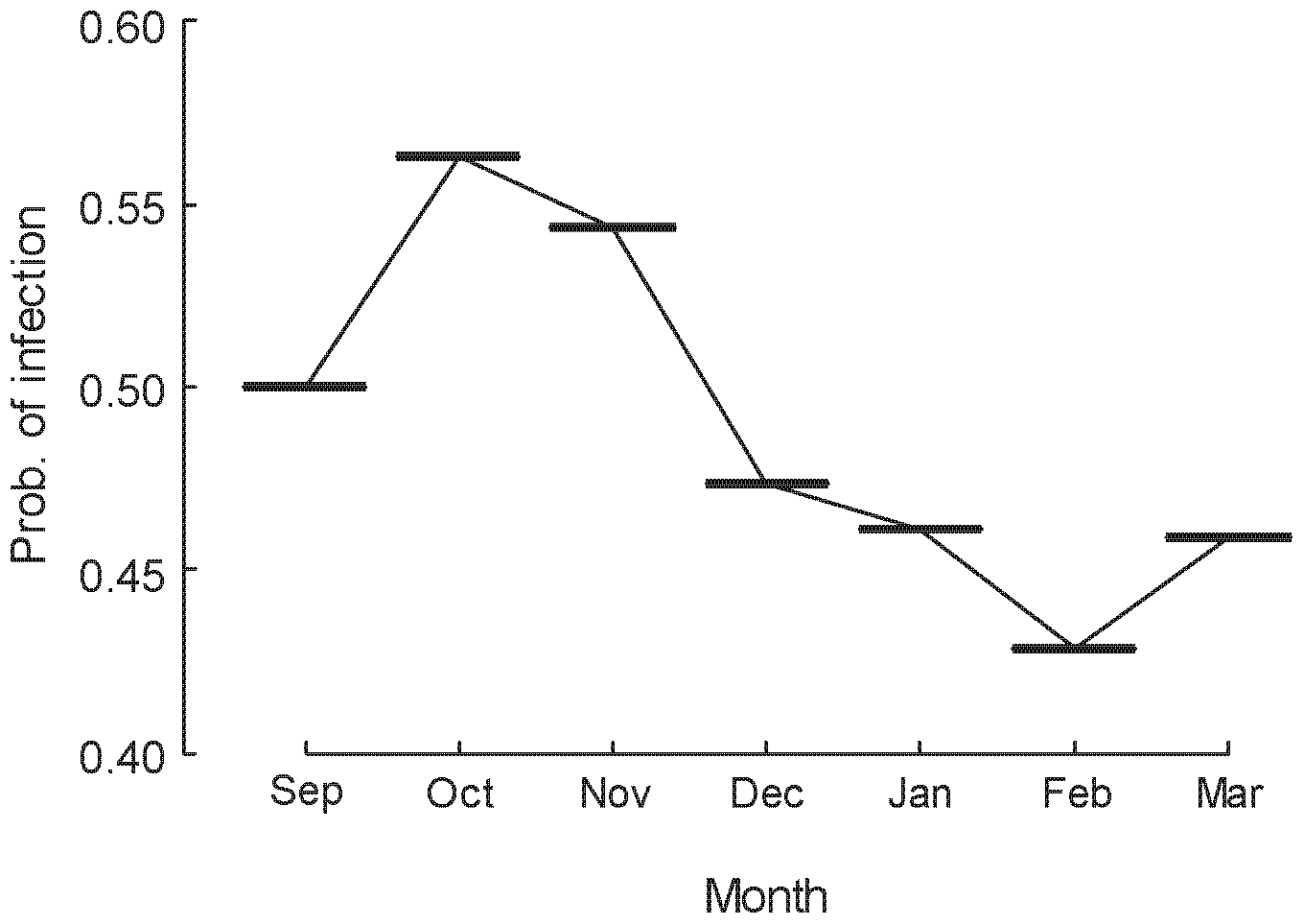
Partial dependence plot from the Random Forest (RF) framework showing the relationship between probability of *Bd* infection and month of the active season for adult male *L. pearsoniana*. This seasonal pattern of infection is now considered typical in forest frogs in subtropical south-east Queensland (see e.g., Kriger and Hero 2007).

The seasonal effect of changes in these variables is well captured in the partial dependence plot of month; with all data taken together, probability of infection peaked in October and November (Spring) and rapidly declined as summer progressed (Fig. 4). Analyses incorporating the process-based model predictions (GI_30_) are presented below.

### Process-based Model Predictions (GI_30_)

**Figure 5:**
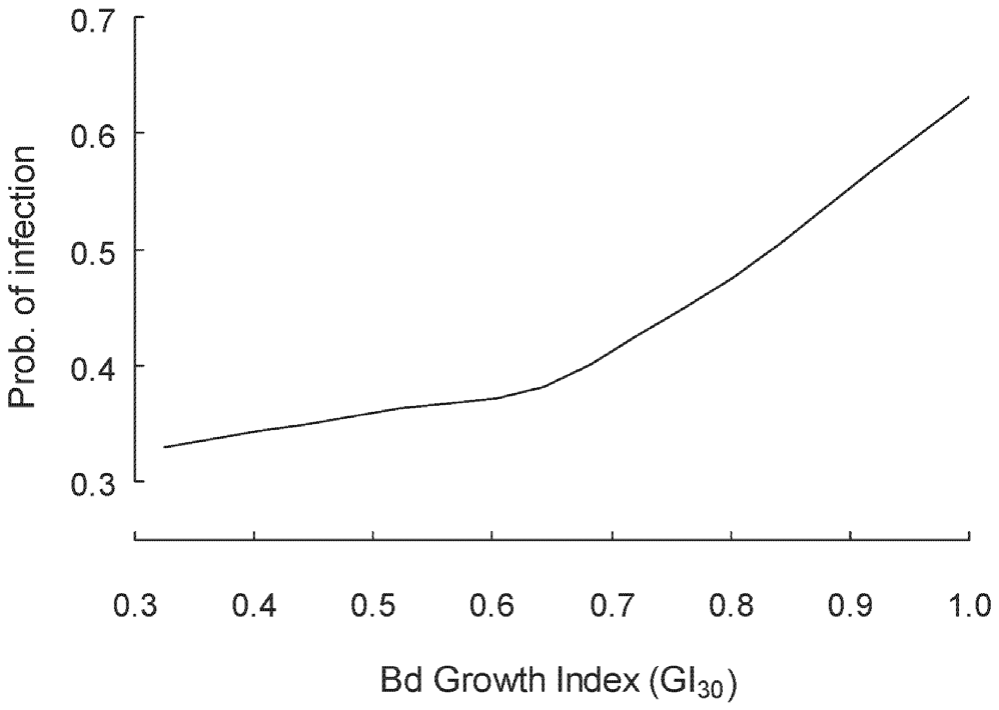
Partial dependence plot from the Random Forest (RF) framework showing the relationship between the probability of *Bd* infection and GI_30_ (simulated pathogen growth in the 30 days prior to sampling, from the CLIMEX process-based model) for adult male *Litoria pearsoniana.* The response line is a lowess smoother. Data were derived from four field sites across three years of sampling (2006–2009). In a model containing the 30 day growth index (GI_30_), body length (SUL) and Rain (mm on the day of sampling), positive PCR results for *Bd* infection were correctly predicted in ∼72% of cases (see results).

In a RF model in which the variables potentially representing simple differences in prevailing weather conditions (T.Max_30_, RH.minT_30_, month, year, season, site) (see above) were replaced with the simulated growth index (GI_30_) for *Bd* from the process-based model (i.e., containing only the GI_30_, SUL and Rain on the day of sampling), there was virtually no loss in classification accuracy of the model (PCC = 67.4%) (Kappa = 0.350, 95%CI = 0.294–0.406). Specificity (% of negative tests correctly classified) was 63.3%, while sensitivity (% of positive tests correctly classified) remained reasonably high at 71.9%. In a test of Prediction 3 from our weather-linked *Bd* proliferation hypothesis, the relationship between probability of infection and the GI_30_ (simulated pathogen growth) was positive (Fig. 5; the relationships for SUL and Rain on the day of sampling in this model were very similar to Fig. 3 and are not reproduced).

### Predictions 1, 2 and 3 Derived from the Weather-linked *Bd* Proliferation Hypothesis

**Figure 6:**
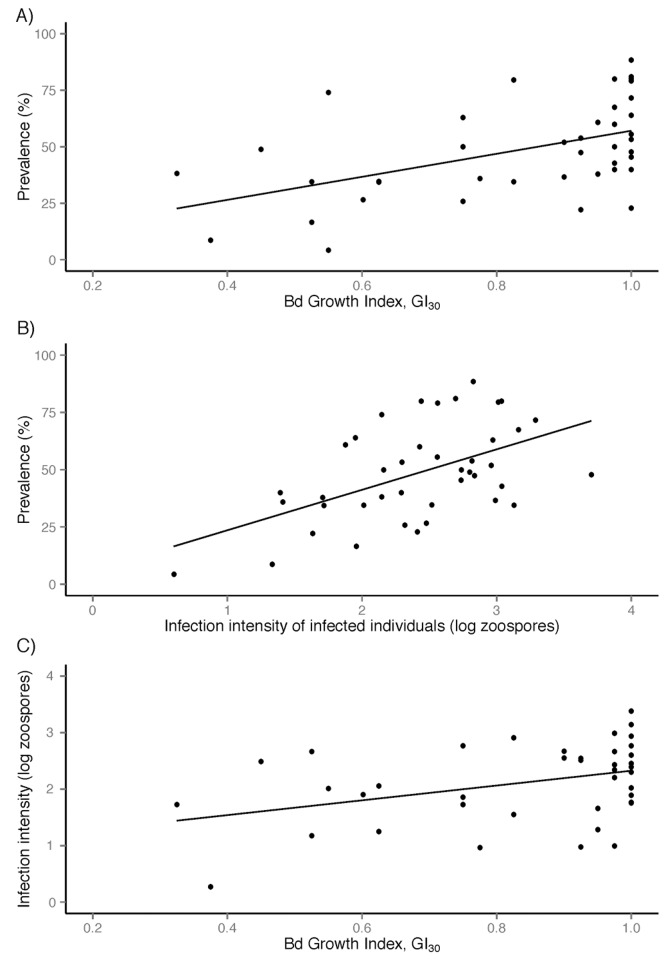
Under the ‘weather linked *Bd* proliferation hypothesis’, chytridiomycosis dynamics are largely driven by pathogen proliferation (growth) under suitable climatic conditions. This hypothesis predicts that modelled pathogen growth (GI_W_, averaged over the 30 days prior to sampling; GI_30_) should be positively related to a) disease prevalence in the population (R^2^ = 0.247, F_1,39_ = 12.79, p<0.001) (Prediction 3) and c) infection intensity of infected individuals (R^2^ = 0.186, F_1,39_ = 8.91, p = 0.005) (Prediction 1) because frogs are ectothermic growth media and disease/transmission dynamics will be dependent on the number of dispersing zoospores. This hypothesis thus also predicts that b) population prevalence should be positively related to infection intensity of infected individuals (R^2^ = 0.284, F_1,39_ = 15.49, p<0.001) (Prediction 2).

As noted above, we found a positive relationship between simulated pathogen growth (GI_30_) and probability of infection (Prediction 3; Fig. 5). This relationship translated well to describing disease prevalence in the population: focussing on data from the four main study sites and the model species *L. pearsoniana*, GI_30_ was positively related to disease prevalence (R^2^ = 0.25; F_1,39_ = 12.79, p<0.001) (sample sizes all ≥15 frogs tested per estimate, mean = 33 frogs tested per estimate) (Fig. 6a). In a test of Prediction 2, infection prevalence in a sampled population was positively correlated with mean intensity of infection (log zoospores) (n = 40), both when all frogs were considered (R^2^ = 0.49) and when only infected frogs were considered (R^2^ = 0.28; F_1,39_ = 15.49, p<0.001) (Fig. 6b). Finally, in a test of Prediction 1, GI_30_ was also positively related to mean infection intensity (log zoospores; R^2^ = 0.19; F_1,39_ = 8.91, p = 0.005) (Fig. 6c). Neither RH.minT_30_ nor T.max_30_ was linearly related to prevalence or infection intensity (data not shown, but see Fig. 3).

## Discussion

The key finding of this study is that we document strong support for the weather-linked *Bd* proliferation hypothesis for endemic chytridiomycosis, which suggests that *Bd* proliferation (population growth) in the wild is at least partly governed by prevailing weather conditions and is a key driver of infection/transmission dynamics in wild, endemically infected amphibian populations. This provides a useful tool with which to enhance surveillance strategies (e.g., by focusing delimitation surveys to temporally and/or geographically coincide with high predicted *Bd* growth and hence enhanced detectability) and to begin making projections about disease outbreaks in the past (e.g., interpreting patterns of historical declines [13], [16], [55]), present (e.g., real-time preparedness and response [20], [40], [56]) or future (e.g., climate change scenario investigation [57]). These applications form the basis for our ongoing work (KAM and LFS unpubl. data) and are not explored further herein. Unlike previous studies that have retrospectively and descriptively used ‘thermal optima’ type models to qualitatively interpret infection patterns, we present a novel test of our hypothesis by developing a weather-linked *Bd* proliferation model, which simulates pathogen growth under varying weather conditions. We then explicitly probed the utility of this model for the prediction of *Bd* infections in wild-caught amphibians sampled across multiple sites and years.

For this process-based model, we integrated *Bd’s* measured responses to temperature and inferred responses to moisture availability to produce a weekly growth index (GI_W_) that can be calculated on any climatic dataset with measurements of temperature (maxima and minima), rainfall and relative humidity (maxima and minima). In our case, we ran the model on daily weather data at our field sites (interpolated from nearby weather stations) to simulate *Bd’s* growth potential in the 30 days prior to sampling (GI_30_). We used this index as an explanatory variable in models describing infection patterns in a model species and hypothesised that under a weather-linked *Bd* proliferation hypothesis, which links pathogen proliferation to disease outbreaks, the GI_30_ should be positively related to disease prevalence (Prediction 3) and intensity of infection (Prediction 1), which themselves should be positively related (Prediction 2).

We found strong support for these predictions. In the RF analysis, infections were correctly predicted ∼72% of the time using a full arsenal of climatic and other variables to inform the model. When all of the variables expected to contribute to climatic suitability for the growth of *Bd* were replaced with the single GI_30_ variable (but retaining the other important, non-climatic factors body size and rainfall on the day of sampling), the model showed comparable performance in predicting infection status (and indicated GI_30_ was the most important variable for predicting infection status). Furthermore, unlike the original climatic variables, which showed non-linear humped relationships with infection probability, the GI_30_ was positively related to infection probability in the RF framework (note that the significant GI_30_^2^ term in the GLM in Table A6.2 in Appendix 6 in Materials S1 also indicates some curvature in the response, which is consistent with Fig. 5); thus, higher predicted growth was positively associated with higher infection probability. Notably, there was a striking similarity between the temperature values used in the process-based model (Figure A1a in Appendix 1 in Materials S1) and the independent field-derived response of infection to average maximum temperature (Fig. 3).

Similarly, in the corroborating logistic modelling framework (Tables A6.1 and A6.2 in Appendix 6 in Materials S1), GI_30_ provided additional explanatory information to the minimum adequate model (MAM) developed from the raw climatic variables. However, while the GI_30_ was clearly important for explaining variation in the data, it was not a sufficient replacement for all of the other variables encoding climatic information. While the significant effect of month was lost in the MAM containing GI_30_, maximum temperature, humidity, year and site still contributed additional explanatory information. This is not surprising given that these non-mechanistic variables are likely to capture non-climatic factors not included in our models that may influence infection, such as host density for example, and given the many leaps that must be made in translating experimentally derived data on pathogen growth to a predictive model that (uncertainly) aims to quantify only a single link in the epidemiological chain. This may also explain the moderate level of variance explained by the final GLM. Many other factors can be expected to corrode this translation when considering patterns of infection as the response, not to mention other metrics such as mortality, population decline or extinction, which are potential responses to infections that have their own strongly modifying predictors over and above infection patterns such as host life-history and ecology [2], [58], [59].

Nevertheless, GI_30_ was positively related to both prevalence within sampled populations (Prediction 1) and intensity of infection of individuals (log zoospores) (Prediction 3), which were themselves strongly and positively correlated (Prediction 2). Stopping short of incorporating mortality and population declines, which according to other recent studies are also intimately influenced by *Bd* proliferation [15], these are the patterns we predicted to see under a weather-linked *Bd* proliferation hypothesis for endemic chytridiomycosis. We have thus developed a potentially predictive and transferable tool for endemic chytridiomycosis that has been tested and found informative for describing patterns of infection in real-world data from within an area considered to be highly suitable for the persistence of *Bd* [20].

### Complicating Factors

The multi-species, single site analyses from Peter’s Creek showed that infection probability is nevertheless highly complex even within a single study site, generally varying among species, years and seasons, and potentially sex and age (Appendix 2 in Materials S1). In some cases the trends were similar (e.g., prevalence in Spring was generally higher than in Summer in *L. pearsoniana* and *L. wilcoxii*; prevalence was highest in the second field season for all species), but in other cases the patterns of infection were unique and unexpected (e.g., a sharp peak in prevalence in *L. chloris* in Summer of the second year). When predicting the impacts of chytridiomycosis on amphibian communities, the weather-linked *Bd* proliferation hypothesis in isolation may thus be a poor predictor of patterns of species infections, declines or extinctions. This is consistent with numerous studies that have demonstrated that the susceptibility and impacts of *Bd* infections in amphibian communities is inherently non-random [1]–[3], [58], [59]. This exemplifies the difficulty of producing predictive epidemiological models for chytridiomycosis in systems comprising multiple susceptible hosts that may differ in any number of important ways, including behaviour, microhabitat use, susceptibility, immunity and demography to name a few. For example, environmentally-dependent host responses may be equally or more important than the effect of environmental factors on the proliferation of *Bd* in regulating infections (e.g., see [32], [60], [61]), and this may differ between species, in which case we might expect some departure from the results we report here (e.g., see [62]). Unravelling the contribution of these additional factors to disease dynamics and building more comprehensive predictive models for amphibian communities or for single species remains a research priority.

Despite this complexity, the commonly reported effect of season was evident in the frog community, particularly in the most well sampled species *L. pearsoniana*, with infections in Spring being more prevalent than in Summer. Similarly, there was a consistent annual trend, whereby all species were more likely to be infected in the second year than in the first or third, giving rise to the suggestion that endemically infected amphibian communities may experience relatively ‘good’ or ‘bad’ chytrid years and seasons. In this study, we show that weather-linked *Bd* proliferation may be highly informative for interpreting and predicting such seasonal or annual infection patterns.

Once major risk factors were accounted for (i.e., species, sex), infection patterns were strongly influenced by prevailing and even daily weather conditions in a susceptible, well sampled model species. Notably, rain on the day of sampling resulted in decreased probability of returning a positive test result; however, rainfall in the 7, 15 and 30 days prior to sampling did not show clear relationships with probability of infection and were not included among the most important variables in the RF analysis for predicting infection status. This is an important and novel finding; while other variables appear more important for predicting monthly and seasonal changes in infection pattern, rain on the day of sampling appears an idiosyncratic or nuisance sampling variable that directly decreased the probability of returning a positive result via some more immediate effect. The mechanism could be simple washing away of zoospores inhabiting sloughing skin cells on the surface of the animal, a suggestion supported by experimental evidence that hosts inhabiting slow moving or still water may have increased infection intensities via the accumulation of infective zoospores (Tunstall unpub. data, cited by [63]). In a *post hoc* test of this hypothesis, we found that zoospore equivalent count indeed responded to rain on the day of sampling (as well as GI_30_), in a similar fashion to the probability of infection (data not shown). This issue thus requires further investigation before swabs taken in the rain can be interpreted with confidence.

In terms of raw climatic factors, in contrast to some studies we found no indication that the effect of radiation (a proxy for e.g., UVB) on infection was as pronounced as other variables in the models (e.g., [62], [64]). Nor are our results consistent with findings suggesting that patterns of temperature-dependent *Bd* growth on frogs is opposite to that obtained from culture [60]. The seasonal infection pattern was best predicted by changes in average maximum temperature and relative humidity in the 30 days prior to sampling. However, the responses characterised between these weather variables and the probability of infection were non-linear; for example, there was evidence to suggest that both low and high maximum temperatures result in reduced disease incidence. In isolation, this provides support for ‘thermal optima’ type hypotheses and goes some way toward empirically integrating contradictory linear responses between temperature and infection reported in previous studies (e.g., [6], [14]). Yet, this is clearly an oversimplification; factors other than temperature, including moisture, are also important and the epidemiological links between climate, pathogen growth, host susceptibility and host response all require consideration before increased disease incidence can be translated directly into amphibian mass-mortalities, population declines or extinctions [5], [13], [16], [19], [55], [65]. Despite this, we suggest that the weather-linked *Bd* proliferation model represents a useful baseline on which these other factors may be overlain, providing new insights about how we might best sample for *Bd* or implement management actions with respect to changes in prevailing weather conditions and longer-term changes in climate.

## Supporting Information

Materials S1 **Appendices 1–6.** Appendix 1, Details of the ‘weather-linked Bd proliferation model.’ Appendix 2, Multi-host infection patterns at Peter’s Creek. Appendix 3, Preliminary tests of the Random Forests modelling framework. Appendix 4, Extent of infection in the model species *Litoria pearsoniana.* Appendix 5, Preliminary analysis of the *Litoria pearsoniana* dataset from the four main study sites. Appendix 6, Generalised linear model corroboration.
(PDF)
