# Supplementary material for: Whether the Weather Drives Patterns of Endemic Amphibian Chytridiomycosis: A Pathogen Proliferation Approach
Source: PLoS One. 2013 Apr 17;8(4):e61061. doi: 10.1371/journal.pone.0061061 (PMC3629077; doi:10.1371/journal.pone.0061061)
Supplement: Materials S1 — Appendices 1–6. Appendix 1, Details of the ‘weather-linked Bd proliferation model.’ Appendix 2, Multi-host infection patterns at Peter’s Creek. Appendix 3, Preliminary tests of the Random Forests modelling framework. Appendix 4, Extent of infection in the model species Litoria pearsoniana. Appendix 5, Preliminary analysis of the Litoria pearsoniana dataset from the four main study sites. Appendix 6, Generalised linear model corroboration. (PDF) [file pone.0061061.s001.pdf]

Materials S1: Appendices 1-6

Whether the weather drives patterns of endemic amphibian chytridiomycosis: a pathogen proliferation approach

Kris A. Murray[1*], Lee F. Skerratt[2], Stephen Garland[2], Darren Kriticos[3], Hamish McCallum[4]

[1]EcoHealth Alliance, 460 W34th St, 17th Floor, New York, New York, 10001

[2]School of Public Health, Tropical Medicine and Rehabilitation Sciences and the Amphibian Disease Ecology Group, James Cook University, Townsville, Queensland 4811, Australia

[3]CSIRO Entomology and the Cooperative Research Centre for National Plant Biosecurity, GPO Box 1700, Canberra, ACT 2601, Australia

[4]School of Environment, Griffith University, Nathan Campus, Queensland, 4111, Australia

[*]Author for correspondence: murray@ecohealthalliance.org

APPENDIX 1 - **Details of the 'weather-linked Bd proliferation model'**

Figure A1. a) Bd growth in culture from the data of Piotrowski et al. (2004). The data are fitted with a quartic polynomial (black line). CLIMEX standard procedure is to use a plateau-shaped thermal function to approximate the typical quadratic response function such as that observed here. We instead estimated T_0 and T_3 (growth limits) from where the fitted polynomial function intersected the x axis to the nearest half degree (3°C and 29°C, respectively). The lower and upper optimal performance temperatures T_1 and T_2 (between which growth is optimal) were estimated by combining observations from Piotrowski et al. (2004) and Woodhams et al. (2008). Lower optimal temperature (T_1) was adjusted to 10°C to accord with Woodhams et al. (2008) who demonstrate that Bd maintains relatively high population growth at low temperatures via life-history trade-offs, which see increased zoospore production as sporangium maturation rate decreases. The upper optimal temperature (T_2) was set to 25°C to accord with Piotrowski et al. (2004). The dotted line depicts these critical threshold temperatures as they relate to the data of Piotrowski et al (2004) (note that in CLIMEX growth potential at $T_0 = T_3 = 0$ and $T_1 = T_2 = 1$); b) Distribution of the 821 sites (n = 10183 specimen records) represented in the dataset of Murray et al. (2010) used to estimate the moisture response parameters for the CLIMEX mechanistic model; c) the predicted environmental suitability (as represented by the CLIMEX Ecoclimatic Index) for the persistence of Bd given the temperature and moisture parameters used in generating the growth index (GI). The fit of the model predictions with the observed global distribution of Bd (courtesy of Matt Fisher and Dede Olson http://www.spatialepidemiology.net/bd/), indicates that a reasonable moisture response curve was found (via iterative fitting) for the derivation of the growth index, GI (see main text and Zalucki and van Klinken 2006 for further details).

a)

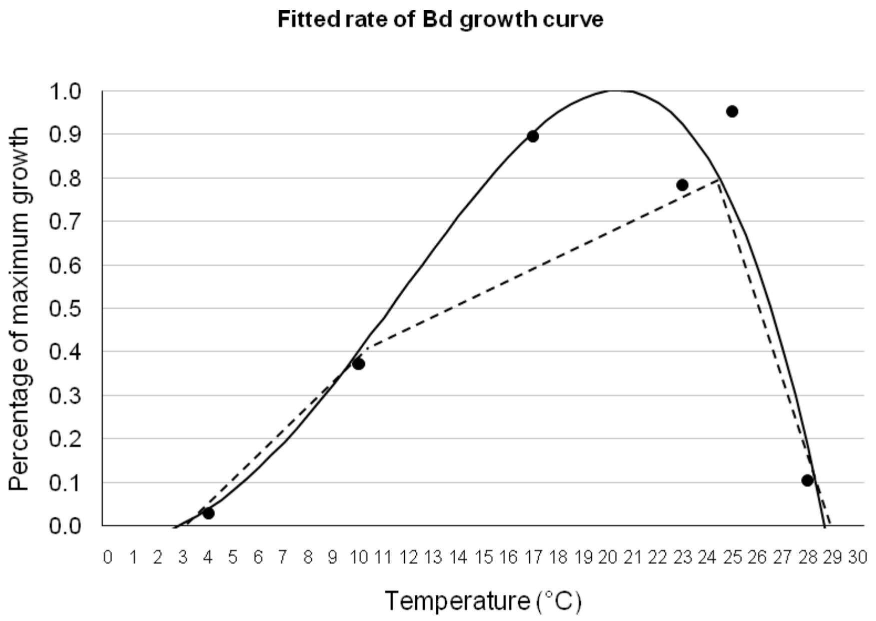

b)

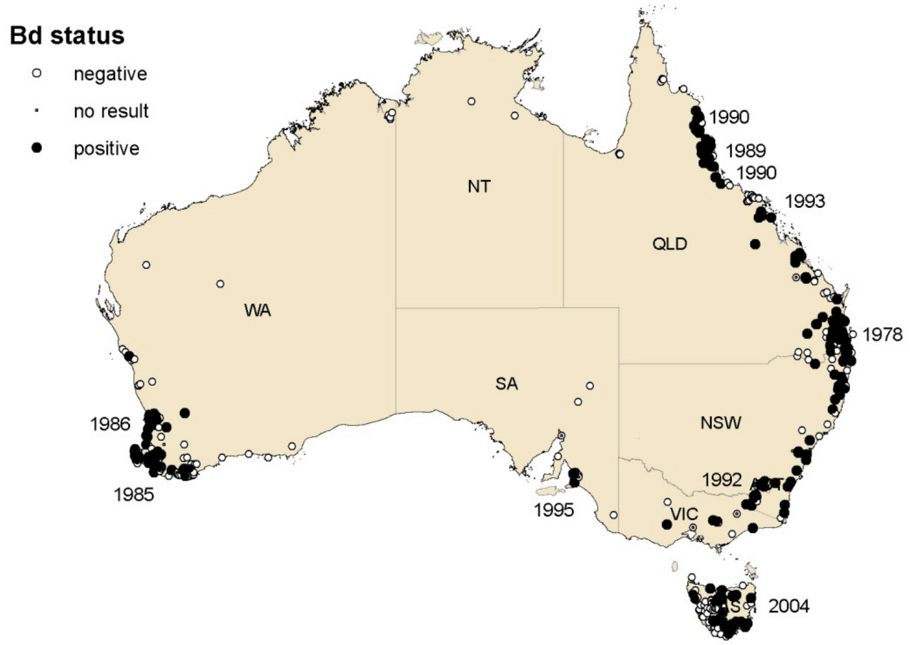
Bd status
negative
no result
positive
NT
QLD
WA
SA
NSW
VIC
1990
1989
1990
1993
1978
1986
1985
1995
1992
2004

c)

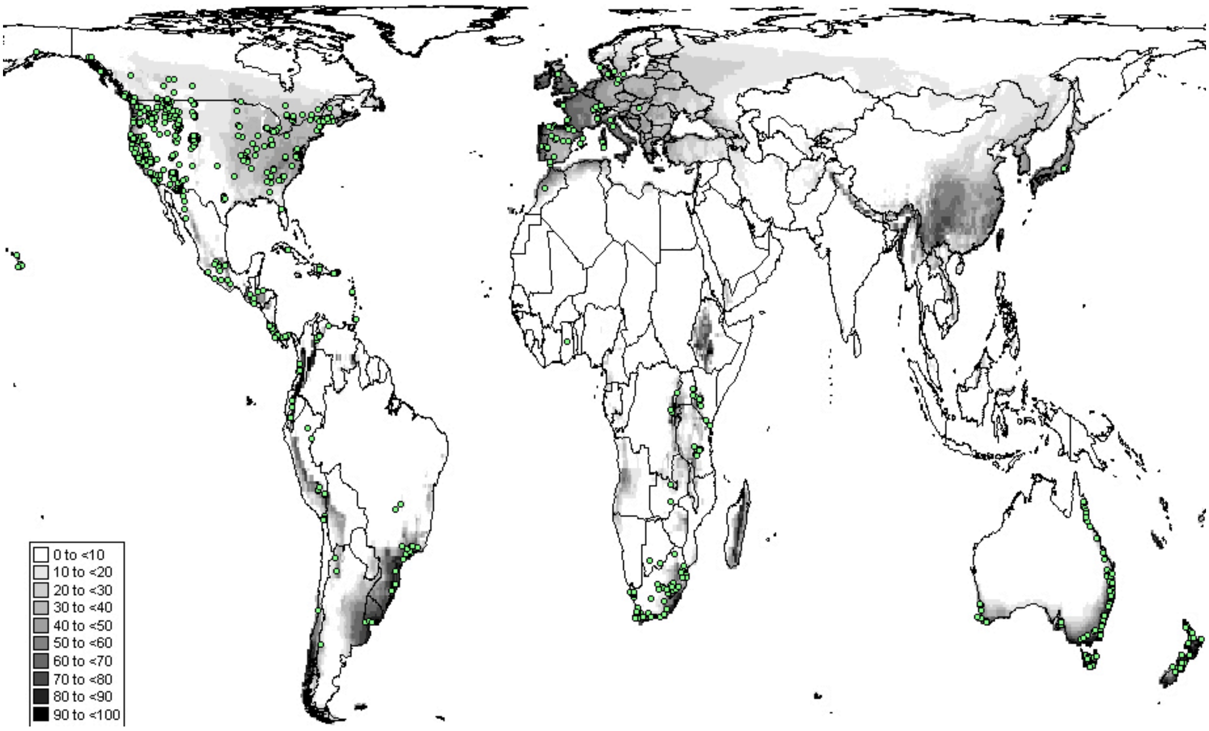

0 to <10
10 to <20
20 to <30
30 to <40
40 to <50
50 to <60
60 to <70
70 to <80
80 to <90
90 to <100

APPENDIX 2 - **Multi-host infection patterns at Peter's Creek**

Single-site, multi-host infection patterns

Nine species were encountered at the main Peter's Creek study site over the duration of the study. We made 1431 captures and swabbed 1072 animals to investigate infection dynamics at this site, the difference representing recaptured animals within field trips that were not re-swabbed. Overall apparent disease prevalence in the frog community was 37.5% (95%CI = 34.6 – 40.5%), but this varied among species (Table A2). Of the nine species encountered, five were detected with Bd infections. Four of the five Bd-positive species were relatively well sampled, comprising 98% of captures (*Adelotus brevis, Litoria pearsoniana, Litoria chloris* and *Litoria wilcoxi*i) so further analyses were restricted to these species.

Table A2. Species mean prevalence across three field seasons at Peter's Creek

					95%CI	
**Species**	**Captures**	**Swabs**	**Bd pos**	**Prevalence**	**lower**	**upper**
Adelotus brevis	29	23	16	0.696	0.471	0.868
Litoria pearsoniana	942	688	312	0.453	0.416	0.492
Mixophyes fasciolatus	13	12	4	0.333	0.099	0.651
Litoria chloris	254	216	47	0.218	0.164	0.279
Litoria wilcoxi	182	123	23	0.187	0.122	0.267
Rhinella marina	6	6	0	0.000	0.000	0.459
Litoria peronii	2	2	0	0.000	0.000	0.842
Litoria fallax	1	1	0	0.000	0.000	0.975
Litoria verrauxii	2	1	0	0.000	0.000	0.975
**Total**	**1431**	**1072**	**402**	**0.375**	**0.346**	**0.405**

In a logistic model, a three-way interaction effect on infection between species, year and season (Spring, Summer) was supported (Δdev = 13.61, df = 6, p = 0.034). Species were subsequently treated separately below.

Infection in *A. brevis*

Adelotus brevis had the highest overall infection prevalence (69.6%; 95%CI 47.1-86.8%) of the species encountered during the study (Table A2. Fig. A2), with between 3-10 times the odds of being infected than the other species (*L. pearsoniana* OR = 2.71, 95%CI 1.13-7.24; *L. chloris* OR = 8.03 95%CI 3.20-22.29; *L. wilcoxii* OR = 9.61, 95%CI = 3.64-28.03). Few data were available for temporal analysis for *A. brevis*, but a logistic model indicated support for effects on infection of sex (Δdev = 6.592, df = 1, p = 0.010) and an interaction between year and season (Δdev = 7.011, df = 2, p = 0.030). Little sense can be made of these effects given the very small sample sizes for individual categories, but males had higher overall prevalence (77.8%, 95%CI = 52.4 – 93.6%) than females (40.0%, 95%CI = 5.2 – 85.3%), prevalence was higher in summer than spring for two of three study years (which was in contrast to the other species) and prevalence was highest in the second field season (consistent with the other species).

Infection in *L. pearsoniana*

L. pearonsiana had a high mean prevalence across the study (45.3%; 85%CI = 41.6-49.2) and was 2.97 (OR 95%CI = 2.10-4.29) times more likely to be infected than *L. chloris* and 3.58 (OR 95%CI = 2.26-5.91) times more likely to be infected than *L. wilcoxii*. It was also the most frequently encountered species, comprising 65.8% of all captures and 77.6% of all Bd-positive results at Peter's Ck over the three years of the study. In a logistic model, there was support for main effects on infection of the three variables year (Δdev = 51.96, df = d, p < 0.001), season (Δdev = 14.60, df = 1, p < 0.001) and sex (males, females, juveniles; Δdev = 9.31, df = 2, p = 0.010), but no interaction effects were supported. The sex effect appeared due to a small number of juveniles (of unknown sex) having lower overall prevalence (14.2%, 95%CI = 1.8-42.8%) than adults (males 46.4%, 95%CI =42.3-50.4%; females

43.1%, 95%CI = 30.8-56.0%) such that the sex effect disappeared when juveniles were excluded from the model. The year effect was due to the second year of the study having a significantly higher infection prevalence (62.7%, 95%CI = 56.0-69.1%) than in both the first year (26.2%, 95%CI = 20.1-32.9%; Odds Ratio = 4.73, 95%CI = 3.11-7.25), and the third year (46.9%, 95%CI = 40.6-53.2%; OR = 1.90, 95%CI = 1.32-2.75). The third year also exhibited significantly higher prevalence than the first year (OR = 2.48, 95%CI = 1.67-3.74). The strong seasonal effect revealed that infections were nearly twice as likely in Spring (53.4%, 95%CI = 48.2-58.7%) compared to Summer (37.3%, 95%CI = 31.9-43.0%; OR = 1.92, 95%CI = 1.41-2.63) in this species (Fig A2a).

Infection in *L. chloris*

L. chloris had relatively low mean infection prevalence (21.8%, 95%CI = 16.4-27.9%), significantly lower than *A. brevis* and *L. pearsoniana* (see above) but not different to *L. wilcoxii*. In a logistic model, there was support for an interaction effect on infection between year and season (Δdev = 7.732, df = 2, p = 0.021) but no evidence for an effect of sex. This interaction appeared due to an unusual spike in prevalence in Summer in the second year of the study, at which time *L. chloris* not only had higher prevalence than in the preceding Spring (a trend reversal compared to the other two years in this species and compared to the consistent seasonal trend in both *L. pearsoniana* and *L. wilcoxii*) but also the highest prevalence of any species during this sampling period. Like in *L. pearsoniana*, infection in *L. chloris* was much more likely in the second field season compared to the first or third (Fig A2b).

Infection in *L. wilcoxii*

L. wilcoxii had mean infection prevalence similar to *L. chloris*. In a logistic model there was minimal support for all two-way interactions among year, season and sex (all $p < 0.1$). Dropping variables on the basis of a $p<0.05$ criterion, however, resulted in all interactions being lost from the model; the best model retained only a main effect of year (Δdev = 22.435, df = 2, $p < 0.001$). A marginal effect of season (Δdev = 3.382, df = 1, p = 0.066) was dropped in the final step. The year effect was similar to that found in *L. pearsoniana* and *L. chloris*; the second year of the study saw greatly increased infection prevalence (40.0%, 95%CI = 23.8-57.9%) compared to the first year (2.1%, 95%CI = 0.0-11.1%; OR = 26.81, 95%CI = 4.86-680.76) and marginally higher prevalence compared to the third year (20.5%. 95%CI = 9.3-36.5%; OR = 2.53, 95%CI = 0.91-7.48, p = 0.080). Although not significant, the marginal seasonal effect was also similar in trend to *L. pearsoniana*, with frogs having generally greater probability of infection in Spring (23.1%, 95%CI = 13.5-35.2%) than Summer (14.0%, 95%CI = 6.3-25.8%; OR = 1.81, 95%CI = 0.71-4.93, p = 0.250) (Fig A2c).

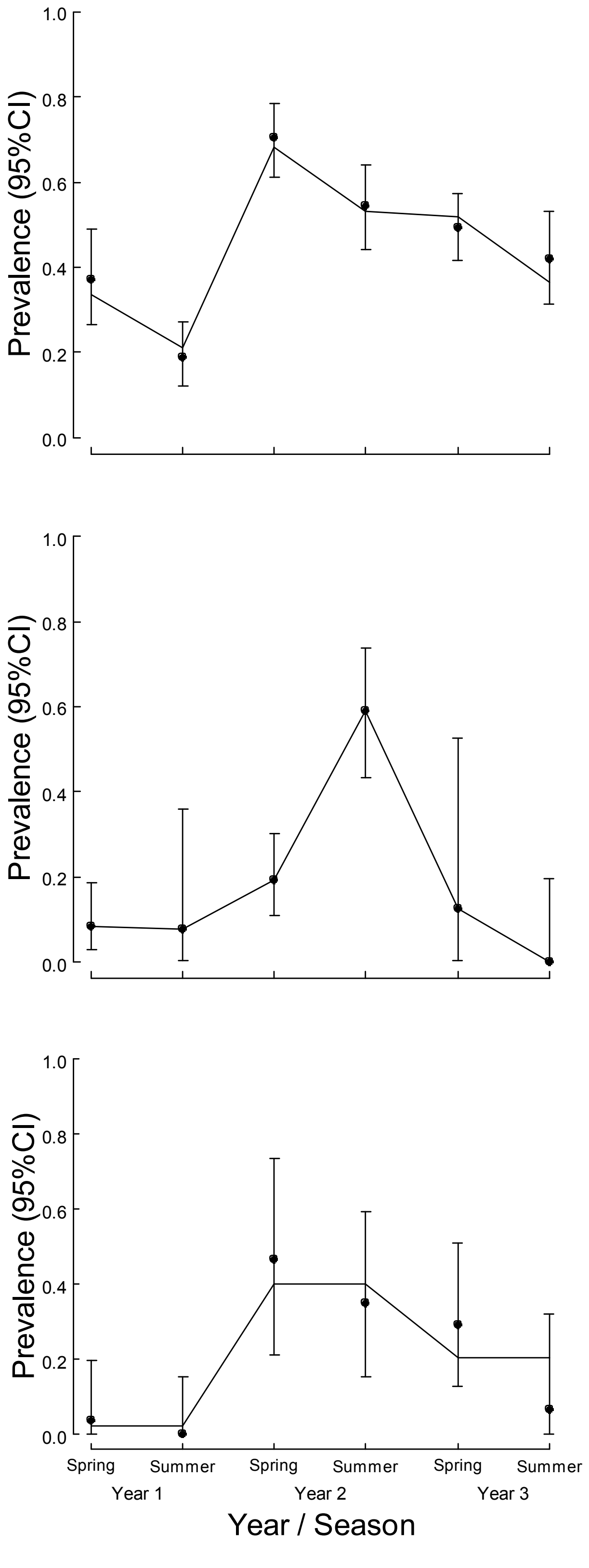

Figure A2. Prevalence patterns in a) *L. pearsoniana,* b) *L. chloris* and c) *L. wilcoxii* across 3 years of study (2006/7-2008/9) at Peter's Ck. The lines are the fitted models for each species (see text).

APPENDIX 3 – **Preliminary tests of the Random Forests modelling framework**

Multi-site, multi-species survey

Before focussing on the model species at multiple sites, the RF modelling approach was tested for consistency with the above results on the 'maximal' dataset, comprising captures of the four main species from all sampled sites across the full course of the study (total of 2097 swabs analysed). The relationships between the predictor variables and the infection response, as indicated by partial dependence plots, strongly mirrored the patterns observed in the previous species by species parametric analyses with respect to the effects of species, year, sex and season (Fig A3). In a starting model that included each of the main variables explored above (species, year, season and sex), the OOB estimate of classification error rate (an internally derived measure of model predictive performance) in the Random Forests analysis was intermediate at 32.1%, meaning that the overall percentage of cases correctly classified (PCC) was 67.9% (1424 of 2097 tests). Specificity (% of negative species correctly classified) was 73.4%; however, sensitivity (% of positive species correctly classified) was lower at 60.2%. RF analysis showed 'fair' performance in predicting infection status as measured using a Kappa test (Kappa = 0.342, 95%CI = 0.302-0.383, where 0 = random prediction, 1 = perfect prediction).

Of the captures used in the 'maximal' analysis, 49.8% came from the main Peter's Ck study site (analysed above). Most (85.7%) of the additional captures outside the main study site were of the focal species, *L. pearsoniana.* Further analyses were thus restricted to this species, sampled from a total of 23 sites (1587 swabs analysed) over a three year period).

.

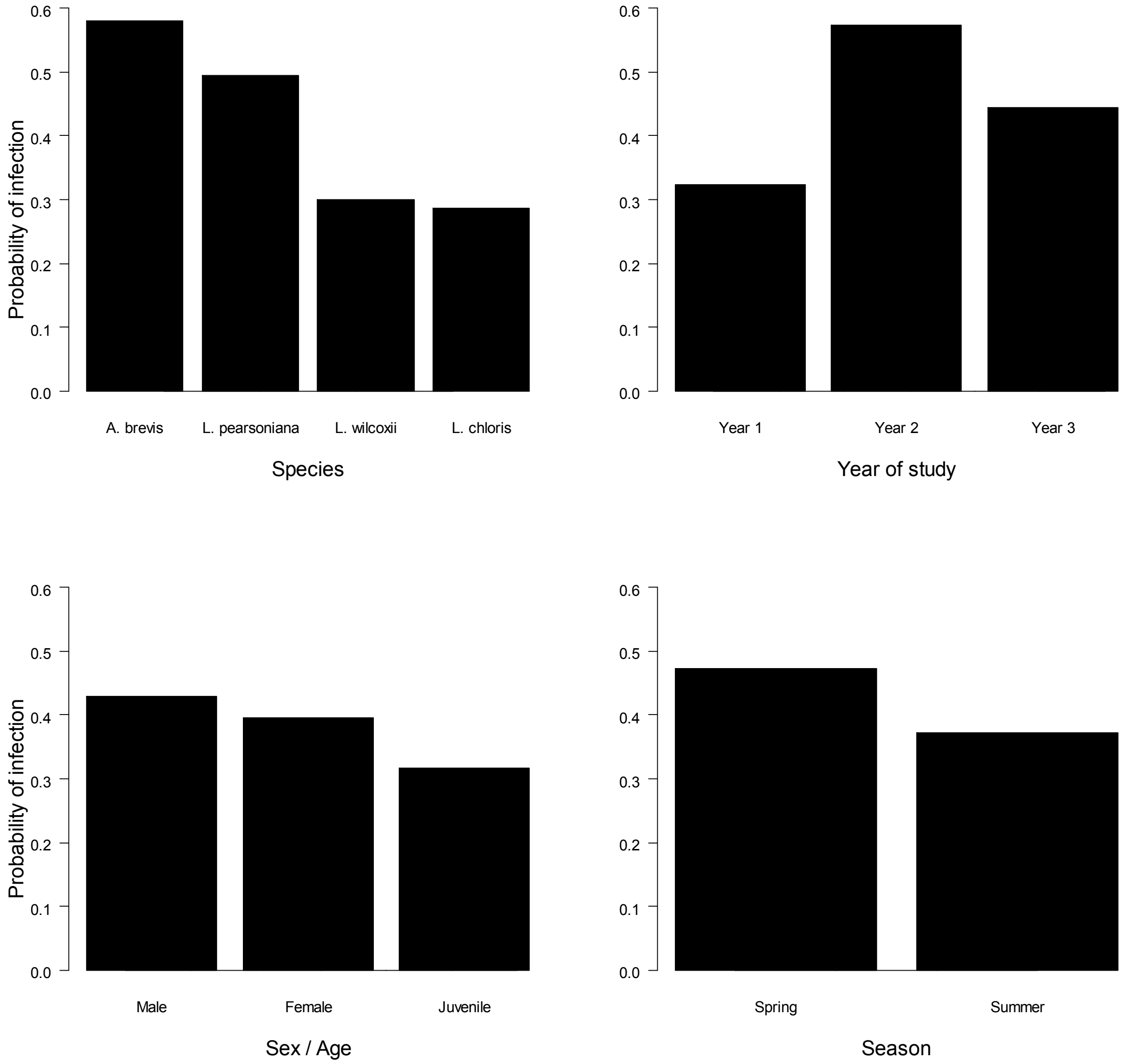

Figure A3. Results from preliminary Random Forests models indicating the effect of four variables (species, year, sex and season) on the probability of an individual returning a positive qPCR result. It can be seen that these results strongly mirrored those described in Appendix 2, in which a clear species, year and season effect was observed.

APPENDIX 4 – **Extent of infection in the model species *Litoria pearsoniana***

Methods

To investigate extent of infection in the model species, an intensive sampling effort across 20 additional populations spanning the geographic range of *L. pearsoniana* was undertaken. Sampling occurred in October of the third year of the study (2008). Study sites were selected based on the data of Parris (2001). Selected study streams were searched until a predetermined number of frogs (n=15) had been captured and swabbed. We anticipated from our surveys at the main sites that prevalence in this species at this time of year would be peaking and therefore high if the site was infected, so our sample size reflected this expectation; our target of 15 animals ensured that we should detect at least 1 positive animal with 99% confidence if prevalence in the population was >25% and sampling approximated random (Digiacomo and Koepsell 1986). Murray et al. (2009) show that detection probability of *L. pearsoniana* is not a function of infection (i.e., infection does not influence likelihood of capture), indicating that these prevalence surveys likely represent a relatively unbiased picture of prevalence among these populations.

Results

In the extent of infection survey (n=201 frogs tested), Bd was detected at all of the 19 sites where >1 animal was tested. Mean prevalence was 71.1% (95%CI = 64.4-77.3%). Of 9 sites at which the target sample size was achieved, mean prevalence was 75.7% (95%CI = 67.8-82.6%), with one site having 100% of frogs in the sample infected (n=15).

APPENDIX 5 - **Preliminary analysis of the *Litoria pearsoniana* dataset from the four main study sites**

Since 88.4% of the data collected on *L. pearsoniana* came from just the four main study sites, more detailed trend analyses were restricted to those sites. In a preliminary analysis on these data, males had marginally significantly higher probability of infection than females (OR = 1.35, 95%CI = 0.94 – 1.96, p = 0.10) and significantly higher infection probability than juveniles (OR = 1.65, 95%CI = 1.21 - 2.27, p = 0.001). Females had non-significantly higher probability of infection than juveniles (OR = 1.22, 95%CI = 0.78 – 1.92, p = 0.42). The remaining analyses were restricted to males only, which comprised 76.4% of the data.

APPENDIX 6 - **Generalised linear model corroboration**

Once the filtering process was complete and an adequate set of predictors was identified with RF (see Methods – Overview of statistical methods), logistic regression was used to confirm the importance of variables remaining in the pruned RF models. Although the machine learning method RF does an excellent job in navigating complex higher dimensional data structures, particularly where a large number of potentially cross-correlated and interactive predictor variables are of *a priori* interest, we included supplementary analyses with more conventional GLMs for the benefit of readers unfamiliar with machine learning methods in ecology. See also Appendix 3.

Methods

The large number of interactions possible in the maximal models could not be fitted, so only the main effects were tested. Curvature in the continuous response variables was considered using quadratic terms.

To test for the influence of the raw climatic variables on infection status, a minimum adequate model (MAM) was first found from a maximal model containing the terms remaining in the pruned RF. To test whether the GI_{30} contributed further explanatory information to this model, it was added as a term to the MAM. To test whether GI_{30} was a suitable substitute for, or redundant to, the other variables potentially describing simple climatic differences in the maximal model, a new MAM was found from the maximal model now containing both the GI_{30} term and the raw climatic predictors.

Results

In a logistic model, the importance of variables remaining in the pruned RF containing raw climatic variables ($T.max_{30}$, $RH.minT_{30}$, Rain on the day of sampling, month, year, site, SUL, season) was confirmed. Single term deletion tests for the terms in the minimum adequate model (MAM, in terms of minimising the AIC) are shown in Table 1. A second model containing only the GI_{30}, SUL and Rain on the day of sampling (analogous to the RF) had significantly poorer fit (data not shown) and as such (unlike in the RF framework) was not an adequate replacement for the more complex model.

The addition of GI_{30} and GI_{30}^2 terms, however, significantly improved the fit of the MAM above (Δdev = 11.89, df = 2, p=0.003) and both terms were independently significant in a single term deletion test (GI_{30} Δdev = 11.49, df = 1, p<0.001; GI_{30}^2 Δdev = 9.73, df = 1, p=0.002), indicating that GI_{30} contains additional explanatory information over and above the raw climatic predictors. This was not surprising since the GI was originally derived from temperature (daily minima and maxima), rainfall (daily) and humidity (daily minima and maxima) data integrated into two key organism-specific indices, temperature and soil moisture, and as such can be expected to contain information not captured by the single raw climatic variables, which were not modelled with interactions.

A new MAM from the maximal model now containing the GI_{30} terms indicated that two climatic variables ($RH.minT_{30}$, $T.max_{30}$) in addition to Year retained explanatory information even when the significant effects of the GI_{30} terms were included (Table 2). Hence, while GI_{30} improved model fit, it was not an adequate 'replacement' for the other climatic variables or year. Month, however, was not retained in this model suggesting that GI_{30} was a suitable and more informative surrogate for the seasonal effect that month has on infection. The overall variance explained in this final model was ~20%.

Table A6.1. Minimum adequate model (MAM) in a logistic regression framework testing the influence via single term deletion of all terms identified as important for predicting infection status in the preceding RF analysis that contained raw climatic predictors. An attempt was made to capture the curvature obvious in the RF framework by including quadratic terms. This model significantly outperformed (in terms of AIC) the simpler model containing just the terms GI_{30}, SUL and Rain.

Deleted variable	Df	Deviance	AIC	LRT	P
<none>		1228.81	1264.81		
$Rain^2$	1	1265.42	1299.42	36.61	<0.0001
Year	2	1266.83	1298.83	38.02	<0.0001
Site	3	1242.25	1272.25	13.44	0.0038
$30dayT.Max^2$	1	1236.66	1270.66	7.85	0.0051
SUL^2	1	1235.95	1269.95	7.14	0.0075
30dayT.Max	1	1235.91	1269.91	7.10	0.0077
SUL	1	1235.78	1269.78	6.97	0.0083
Month	6	1245.54	1269.54	16.73	0.0103
$30dayRHminT^2$	1	1233.59	1267.59	4.78	0.0288

Table A6.2. Minimum adequate model (MAM) in a logistic regression framework testing the influence via single term deletion of all terms identified as important for predicting infection status in the RF framework (i.e., the growth index [GI_{30}] was included in addition to the raw climatic predictors).

Deleted variable	Df	Deviance	AIC	LRT	P
<none>		1227.97	1255.97		
Rain	1	1275.04	1301.04	47.07	<0.0001
30dayRHminT	1	1251.73	1277.73	23.76	<0.0001
Year	2	1255.15	1279.15	27.18	<0.0001
30dayT.Max	1	1250.55	1276.55	22.58	<0.0001
$30dayT.Max^2$	1	1248.95	1274.95	20.98	<0.0001
GI_{30}^2	1	1245.12	1271.12	17.15	<0.0001
GI_{30}	1	1243.62	1269.62	15.66	0.0001
Site	3	1244.4	1266.4	16.43	0.0009
SUL^2	1	1237.97	1263.97	10.00	0.0016
SUL	1	1237.54	1263.54	9.57	0.0020

References

Digiacomo, R. F. and Koepsell, T. D. 1986. Sampling for detection of infection or disease in animal populations. — Journal of the American Veterinary Medical Association 189: 22-23.

Murray, K. A. et al. 2010. The distribution and host range of the pandemic disease chytridiomycosis in Australia spanning surveys from 1956 to 2007. — Ecology 91: 1557.

Murray, K. A. et al. 2009. Impact and dynamics of disease in species threatened by the amphibian chytrid fungus, *Batrachochytrium dendrobatidis*. — Conservation Biology 23: 1242-1252.

Parris, K. M. 2001. Distribution, habitat requirements and conservation of the cascade treefrog (*Litoria pearsoniana*, Anura : Hylidae). — Biological Conservation 99: 285-292.

Piotrowski, J. S. et al. 2004. Physiology of *Batrachochytrium dendrobatidis*, a chytrid pathogen of amphibians. — Mycologia 96: 9-15.

Woodhams, D. C. et al. 2008. Life-history trade-offs influence disease in changing climates: Strategies of an amphibian pathogen. — Ecology 89: 1627-1639.

Zalucki, M. P. and van Klinken, R. D. 2006. Predicting population dynamics of weed biological control agents: science or gazing into crystal balls? — Australian Journal of Entomology 45: 331-344.
